# Steps towards implementing evidence-based screening in family medicine in Ukraine: SWOT-analysis of an approach of multidimensional empowerment

**DOI:** 10.1186/s12875-021-01367-2

**Published:** 2021-01-14

**Authors:** Pavlo Kolesnyk, Thomas Frese, Shlomo Vinker, Ivanna Shushman, Albina Zharkova, Nassir Messaadi, Andriy Kolesnyk, Sabine Bayen

**Affiliations:** 1 Department of Family Medicine and Outpatient Care, Medical Faculty #2, Education Scientific Family Medicine Training Centre, Uzhgorod National University, Uzhgorod, Ukraine; 2 Department of General Practice & Family Medicine, Medical Faculty, Martin-Luther University, Magdeburger Straße 8, 06112 Halle /Saale, Germany; 3 Department of Family Medicine, Tel Aviv University, Tel Aviv, Israel; 4 Department of Family Medicine, Sumy State University, Sumy, Ukraine; 5 Department of General Practice, University of Lille, Lille, France; 6 Department of Medical Pharmacology & Neurology, INSERM UMRS 1172, University of Lille, Lille, France

**Keywords:** Family practice, Research action, Implementation study, Evidence-based screening, Empowerment, Ukraine

## Abstract

**Background:**

The purpose of this study is to forward the implementation of an operational evidence-based state screening program of common diseases in Ukraine, where currently no state-based and evidence-based screening (EBS) exists. EBS should be performed by Family Doctors in a primary care setting and concern prevalent diseases in adults, such as: obesity (BMI), hypertension (BP measurement), diabetes (glycaemia), dyslipidemia (cholesterol/lipids), colon cancer (FOBT/colonoscopy), breast cancer (mammography), STIs (chlamydia, syphilis), HIV, HBV, HCV (i.e. serology or other rapid tests), HPV (swabs), cervical cancer (test Pap). depression (i.e., PHQ-9), and smoking (i.e., Fagerstrom).

**Methods:**

Four needs-based research actions were led among citizens and healthcare professionals, based on multidimensional empowerment. Internal Strengths and Weaknesses of the ongoing implementation process were identified through these studies, whereas external Opportunities and Threats were determined by the present socio-cultural and political context. This SWOT analysis is likely to guide future state-based initiatives to accomplish EBS implementation in Ukraine.

**Results:**

Internal Strengths are the bottom-up multidimensional empowerment approach, teaching of EBS and the development of an internet-based platform “Screening adviser” to assist shared decision making for person-centred EBS programs. Internal Weaknesses identified for the Family Doctors are a heterogeneous screening and the risk of decreasing motivation to screen. External Opportunities include the ongoing PHC reform, the existent WONCA and WHO support, and the existence of EBS programs in Europe. External Threats are the lack of national guidelines, not fully introduced gate keeping system, the vulnerable socio-economic situation, the war situation in the East of Ukraine and the Covid-19 pandemic.

**Conclusions:**

We started EBS implementation through research actions, based on a multidimensional empowerment of citizens, HCP and in EBS pathways involved stakeholder teams, to foster a sustainable operational human resource to get involved in that new EBS pathway to implement. The presented SWOT-analysis of this ongoing implementation process allows to plan and optimize future steps towards a state based and supports EBS program in Ukraine.

## Background

Ukraine is a low-income country with 44 million inhabitants in 2018 [1]. In 2014, a reform of primary healthcare (PHC) was elaborated to replace the prior centralized model. Started in 2018, it is extended to secondary and tertiary levels in 2020 [2]. According to the World Health Organization (WHO), providing healthcare (HC) for everyone with accent on PHC is the invariable key indicator of HC development [3]. The Ukrainian government supports that statement and started the development of PHC [4, 5]. WONCA defines health promotion, prevention, and screening as core tasks of Family Medicine (FM) [6]. Whereas evidence-based screening (EBS) fosters early diagnosis of asymptomatic diseases, enables early treatment, reduces complications rates, and improves citizen’s prognosis [7], the screening effectiveness in economically developed countries is linked to considerable resources provided by the respective governments [8, 9].

Nowadays, the HC systems of post-Soviet countries are not able to support high levels of EBS according to modern Western standards [4, 10]. Ukraine has limited financial resources in general and in particular concerning HC [3]. To investigate the evidence level for the existent national screening recommendations and the possibility to implement EBS, it is necessary to conduct a local field study, taking into account the Ukrainian specific medical, psychological, socio-economic aspects of the target population. Furthermore, the willingness, the financial and infrastructural ability of the state to support an implementation of national EBS programs must be evaluated. Beyond the need of funding for the EBS organization itself, the potency of the government to provide following treatment and rehabilitation must be guaranteed to respect the basic principles of evidence-based medicine (EBM) [11–13].

The previous *“traditional”* system of screening in post-Soviet countries was based on low informative investigations (Table 1).

**Table 1:** Overview of transferable existent international state organized EBS programs

Screened diseases	< 1990 [14]	> 1990 [2]	2020 [15]	Aimed EBS [16]
**Hypertension** [2]	–		+/−	+
*BP*				
All genders				
*> 18y.o.*				
*every 2 years*				
**Smoking status**	–		+/−	+
*Asking*				
All genders > 18 y.o				
*At least once, then when needed*				
**Alcohol use**	–		–	+
*AUDIT questionnaire*				
All genders > 18 y.o				
*At least once, then when needed*				
**Obesity** [2]	–		+/−	+
*BMI*				
All genders > 18 y. o				
*At least once, then when needed*				
**Depression**	–		–	+
*PHQ2–9 questionnaire*				
All genders > 18 y. o				
*At least once, then when needed*				
**Diabetes** [2]	+/−		+/−	+
(if BMI > 25 + other factors)				
*Glucose tests*				
All genders > 18 y. o				
*Every 3 years*				
**Dyslipidaemia**	–		+/−	+
*cholesterol/ lipidogram*				
Mails > 35 y. o				
Females> 45 y. o				
*Every 5 years*				
**Cervical cancer**	+	+	+	+
*Pap-test*	Gyn	Gyn	Not always	
Females >21y.o	investigation	investigation	with Pap test	
*Every 3 years*	Not always		–	
*+ HPV test*	with Pap test	Not always		+
> 30 y. o	–	with Pap test		
***Every 5 years***		–		
**Breast cancer**	–		+/−	+
*Mammography*				
> 40 y.o.				
*Every 2 years*				
**Colorectal cancer**	–		–	+
> 50 y.o				
*FOBT+*	–		–	+
*Rectoscopy*				
*Every 5 years*				
*Colonoscopy*				
*Every 10 years*				
**Lung cancer**	–		–	+/−
Males strong smokers				
*CT scan*				
> 55 y.o once				
**Osteoporosis**	–		–	+/−
*Densitometry*				
*FRAX*				
> 50 y.o once				
**Aortic aneurism**	–		–	+/−
Male smokers				
*Ultrasound*				
> 65 y.o *once*				
**Tuberculosis**	+*		+/−*	–
*Annual chest-X-Ray > 18y.o*				
**ECG >** 40 y.o to screen for early heart changes	+		+	+/−
**Urinalysis** *> 18 y.o (for inflammation and haematuria)*	+		+/−	–
**Biochemical test** *(to screen for liver or kidney abnormalities)*	+/−		+/−	–
**Ultrasound** (people often made self-screening for any changes, visceral cancer and stones. That was not part of mandatory screening, but people preferred to make it and payed for it)	+/−		+/−	–
**Complete Blood count** (to detect anaemia and signs of inflammation or indirect cancer signs)	+		+	–
**STI** [2] *HIV, HBV, HCV*	–		+/−	+

According to the last Ukrainian Ministry of HC legislation [2], the post-Soviet screening protocol was cancelled without being yet replaced by a new one.

In 2011, existent screening-like procedures included mandatory annual check-ups, targeting only the working population, schoolchildren and students, including non-evidence-based investigations. At present, Ukrainian citizens are free to choose any method of screening they consider as appropriate for themselves, such as routine annual blood analysis, urinalyses, chest x-ray, gynecological exam with or without Pap-smear, digital prostate exam etc., whereas some of the expenses are covered through out-of-pocket payments. However, some sub-populations of employees, expected to be in touch with public, are still obliged to undergo the prior protocol [14].

That situation creates disparities in access to EBS. To promote the implementation of a state based and supported EBS program in Ukrainian FM seems to be useful and coherent to overcome some of these disparities. A state supported EBS program in FM is crucial to be performed by primary care physicians, FDs and to be proceeded in FM clinics.

We aimed to plan screening for patients starting from 18 years and older. Therefore, we concentrated on the supposed most prevalent diseases and their screening in the FM practice of Ukraine: obesity (BMI screening) and other diseases/risk factors (smoking, depression – screening using the questionnaire), hypertension (BP measurement), diabetes (glycaemia screening), dyslipidemia (cholesterol/lipid profile screening), colon cancer (fecal occult blood test (FOBT)/colonoscopy), breast cancer (mammography), STIs (tests for chlamydia, syphilis), HIV, HBV, HCV (ELISA or other rapid tests), HPV (PCR of the swabs), cervical cancer (test Pap).

We present here our first steps of implementing EBS in Ukrainian primary care settings.

An action research is ongoing, based on the empowerment of the potentially involved actors in the aimed EBS pathway.

To co-construct the aimed EBS pathway with the future involved actors, we chose a *“bottom-up”* strategy [17], based on an approach of multidimensional empowerment of citizens, FD and other healthcare professionals.

Wallerstein defines empowerment in health as a social action through which persons, communities and organizations, acquire life control by changing their socio-political environment to increase equity and to improve quality of life [18]. The WHO considers empowerment as a prerequisite in health promotion [15].

**Fig. 1:**
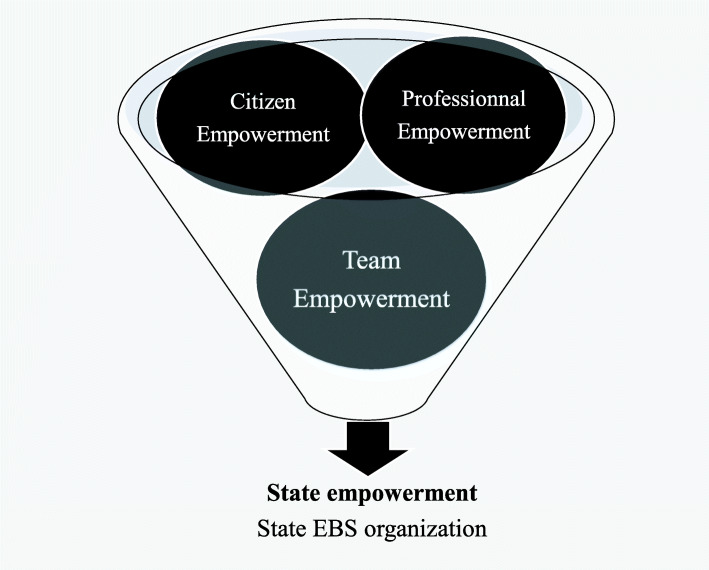
Global empowerment for state organized EBS implementation

The following Fig. 1 illustrates the global concept of the chosen approach to lead that implementation.

The three forms of empowerment will, on the long term, progressively foster the global, state empowerment (SE) to support the ambitious project of EBS implementation in Ukraine.

**Fig. 2:**
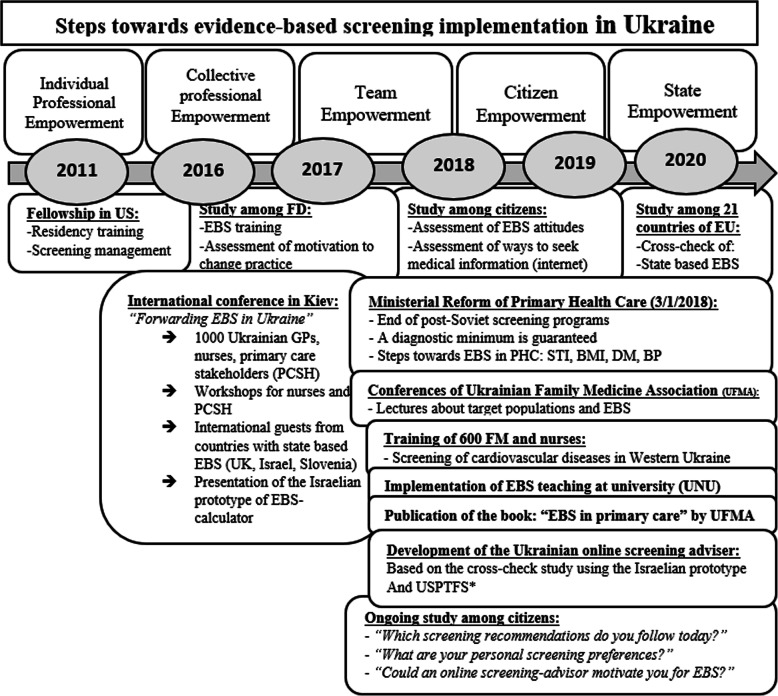
Chronology of the ongoing EBS implementation study

Figure 2 provides an overview of the chronological evolution of that ongoing process.

A SWOT analysis is a strategic planning technique used to help an organization identify Strengths, Weaknesses, Opportunities, and Threats related to business competition or project planning, in our case of an ongoing implementation study [19]. The SWOT assumes that Strengths and Weaknesses are frequently internal, while Opportunities and Threats are more commonly external.

The purpose of our study was to conduct a SWOT analysis of the ongoing implementation process to guide the national health authorities (health ministry and WHO) in future strategic planning of EBS implementation.

## Methods

### Study design

For each population, involved in that EBS implementation, needs-centred interventions were led and are presented in the following Table 2.

**Table 2:** Overview of the study methods

Study	Interactive EBS training [20] *Study A*	Information seeking habits *Study B*	EBS in EU *Study C*	EBS adviser *Study D*
**Aim/approach**	Collective Professional Empowerment	Citizen Empowerment	State Empowerment	Citizen/ Team Empowerment
**Investigation period**	Oct 2016-Oct 2017	Oct 2018-Oct 2019	Oct 2018-Oct 2019	ongoing
**Method**	Mixed method Research action *n* = 20 courses (8 h each)	Quantitative multiple choice	Quantitative multiple choice	Quantitative multiple choice
**Population**	FD	Citizens	National EURACT representatives	Citizens
**Data collection**	Directly before /after intervention+ follow-up at 3 and 12 months	Monocentric, one- shot survey during consultation	Multicentric online survey	Multicentric online survey
**Data analysis**	StataCorp LP®	Excel®	Excel®	SPPS®

### Chronological implementation process of empowerment

An ***individual professional empowerment*** was initiated by the first author**,** an active Ukrainian Family Doctor (FD), working for 20 years in the local municipal FM Clinic as a university trainer. Being interested in an implementation of state organized EBS, he developed EBS strategies for Ukrainian FM [21]. He became the leader [22] of a ***collective professional empowerment*** to increase FDs’ knowledge and motivation to introduce a new EBS system into their clinical practice. To trigger that *collective professional empowerment,* the set of EBS skills training was initiated and successfully conducted among FDs of Western Ukraine which is mainly rural and spread in the mountaineer area where medical care is challenging. During 2016–2017, the FM Training Centre organized interactive training courses on *“Evidence-based steps in prevention, screening and management of cardiovascular risks among citizens in FDs’ practice of Transcarpathian region of Ukraine”,* where over 600 FDs participated. FD’s knowledge was evaluated and changes in their attitudes towards implementing EBS in their clinical practice were assessed, using a score to indicate the degree of motivation to change (low =1–10, moderate =11–20, > 20 = high). Both questionnaires were administered to FDs before, immediately after, 3 and 12 months after the training [20].

***Team empowerment*** was initiated during an international conference entitled *“Forwarding EBS in Ukraine”* in Kiev (conducted twice a year in Kiev by the Ukrainian FM Association) in December 2016. Over 1.000 Ukrainian FD, nurses, delegates of health ministry, and managers of PHC took part. Experts from European countries (UK, Israel, Slovenia) presented their experience in EBS implementation which were supported by their states. Workshops concerning possibilities to develop EBS in Ukraine were organized for FM teams. A web-based program *“EBS-calculator”* developed in Israel was suggested as a prototype for Ukrainian FD’s and citizens’ empowerment [23].

Representatives from the 25 regions have participated in the elaboration of its report, based on their collaborative expertise of that community of healthcare professionals (HCP) involved and interested in EBS and its promotion on a national level. The reports were systematically transmitted to the Ukrainian Ministry of health with practical suggestions to promote EBS.

***Citizen empowerment*** started with an essential *“bottom-up”* study among 237 Ukrainian citizens, who visited the FM clinic. To explore the feasibility of implementing future state based EBS programs in Ukraine (***state empowerment***), we conducted an international, comparative study, including 21 European countries. We aimed to gather examples of common state-based screening programs all over Europe and to correlate them with American guidelines of the most effective recommendations (high levels of evidence: A/B in USPTFS) [16] and often used screening methodologies among them, especially those which involve FDs in primary care settings [16].

## Results

Table 3 allows an overview of the four conducted studies, aiming to foster the EBS implementation process. The study results should be taken into account to plan future EBS strategies, and to improve the quality and outcome of the still ongoing implementation process. Therefore, we will discuss them later under the prism of the SWOT framework.

**Table 3:** Overview of the studies’ main results

Study	EBS training [20] *Study A*	Information seeking habits *Study B*	EBS in EU *Study C*	EBS adviser *Study D*
**Aim/approach**	Collective Professional Empowerment	Citizen Empowerment	State Empowerment	Citizen/ Team Empowerment
**Participants**	*n* = 307 Initial RR: 51.2% RR (training): 100% RR (3 months): 100% RR (12 months): 71%	*n* = 237 M:143; F:94	*n* = 21	At least 1.000
**Main results**	The interactive EBS training increased participants’ knowledge and readiness to change clinical practice. This impact diminished over time but was still evident after 12 months.	The Internet became a main popular source to seek medical information concerning screening and health care, for 39% of men and 48% of women.	Similar recommendations of EBS related to the US preventive guidelines of high grade of evidence (A/B) were composed together to elaborate a list of common diseases/methods of screening.	Expected results: the online EBS adviser will contribute to a more effective and efficient person-centred, shared decision based on evidence

### Study A

The training of FD on EBS aimed to assess their knowledge of EBS and their motivation to use it in their clinical practice [20]. The average level of knowledge was low before training with a mean of correct answers 6.1 (S.D. 1.8) out of 20 but increased significantly after training to 15.3 (S.D. 2.3; *p* < 0,001) and remained higher than baseline at 1 year. The number of highly motivated practitioners was significantly higher immediately after the training than before it, but motivation levels after 1 year decreased substantially.

The proportion of FDs who were highly motivated to change their practice according to new knowledge increased significantly from 9.3% before the training to 37.3% immediately afterwards. Three months later**,** the mean number of correct answers had decreased to 10.23 (S.D. 3.27) but 29.7% of FDs were still highly motivated to change their practice. Twelve months later, the mean number of correct answers was stable 10.34 (S.D. 3.28) comparing with the previous results. Significantly less FDs remained highly motivated (17%) to change their practice.

### Study B

The survey among 237 Ukrainian citizens aimed to determine their information-seeking behaviours regarding EBS and healthy lifestyle.

The mean age of the participants was 36.2 +/− 11.1 years old (25.1–47.3 years); 143 were male (60%). Internet was the main source of medical information for 56 of the men (39%) and 45 of the women (48%). As for the other population, 57 male (39.9%) and 66 female (70.2%) respondents trusted FD’s opinion, 54 men (37.8%) and 12 women (13%) trusted their relatives’ opinion; 13 men (9.3%) and 10 women (10%) followed recommendations of their acquaintances, 19 men (13%) and 6 of the women (6.8%) made their decision by reading literature, etc.

### Study C

**Fig. 3:**
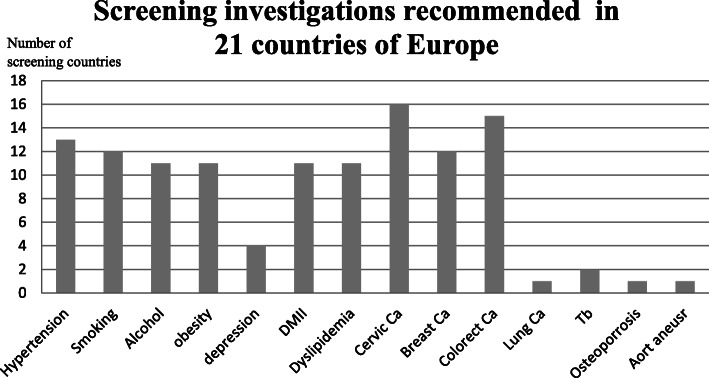
State based and evidence-based screened diseases in 21 European countries

The following 21 countries were included to answer a survey by e-mail via the national representatives of EURACT (European Academy of Teachers): Albania, Belgium, Czech Republic, Denmark, Estonia, France, Georgia, Germany, Greece, Hungary, Italy, Macedonia, Moldova, Netherlands, Poland, Portugal, Russia, Slovakia, Slovenia, Spain, and UK (Fig. 3).

### Study D

A new electronic screening tool for shared decision making was developed. The web-based platform *“Screening Adviser”* (SA) calculates an individual plan of primary preventive measures and screening according to citizen’s personal characteristics. The recommendations are based on mainly used European EBS guidelines. First, a short online survey is developed to evaluate citizen’s opinion concerning appropriate screening: *“Which of the following clinical examinations are appropriate for you as a screening (from the attached list)?”* The citizen can choose several options from the list of screening investigations (including post-Soviet and modern Western EBS recommendations). The second step is the assessment of the citizen’s personal data: age, gender, weight, height, sexual activity status, smoking habits, alcohol consumption. According to the data, the software will develop a personalized list of screening recommendations based on high evidence (level A/B).

The third step invites the citizen to accept or not the EBS suggestions by answering the same question: *“Which of the following exams seem are appropriate for you as a screening (using the same list of investigations mentioned before)?”* The list of investigations, chosen by the citizen as appropriate before and just after using the SA can be compared to determine if and in which way his or her initial choice of EBS was influenced by the given recommendations.

The ongoing multicentric study aims to evaluate which screening investigations citizens request preferentially through the SA*.* Secondary outcomes are as followed: influence of the Internet on their personal choice, personal criteria to consider an investigation as reliable, and how they evaluate and accept, or not, their received results, suggested by the SA. Can the calculated EBS suggestions convince citizens to adhere to a shared EBS decision? The study will be conducted in several municipal FM clinics of Uzhgorod and aims to include at least 1000 adult participants. If the piloting is successful, the adviser will be suggested to be used in different clinics in other regions of Ukraine. At present, a standardized questionnaire, including sociodemographic data, citizen’s initial personal choice of screening investigation, and how they evaluated the EBS suggestions according to their calculated risk profile, is being tested among a small sample. Based on citizens’ answers to questions before and after accepting the personalized list of EBS guidelines, the database will be generated and processed statistically (frequencies of choosing each method of listed screening method before and after getting the advice). Quantitative data are analysed using SPPS software and qualitative data are gathered through a qualitative content analysis.

To initiate future state based EBS programs*,* the international comparison of existing reliable EBS programs in Europe, a first overview and possible transferable future EBS investigations to implement in Ukraine are presented in Table 1.

## Discussion

**Fig. 4:**
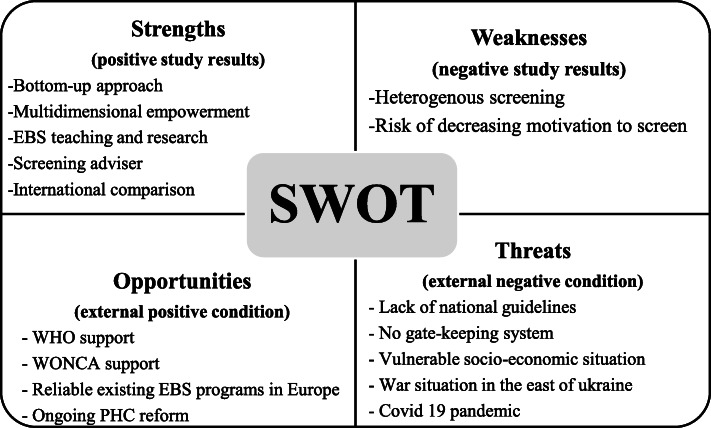
SWOT - Analysis Diagram

Through the SWOT-framework, we will now discuss the internal Strengths and Weaknesses of the current condition of the EBS implementation process and the external Opportunities and Threats given by the current socio-economical-political context (Fig. 4).

### Strengths (internal, positive factors)

The dynamic progressive *individual professional empowerment* fostered the *collective professional empowerment* among peers, which on its turn fostered the *team empowerment* among actors involved in the EBS HC-pathway. A first base of EBS has been implemented.

The accomplished steps of our progressive bottom-up EBS implementation through individual and collective professional empowerment are sustainable since 2016 and led to team empowerment which increases the power and visibility of the community involved in the EBS HC-pathway.

The traditional continuous medical education in Ukraine was based on a descending teaching and no studies about the effectiveness of interactive motivational training methods existed for training Ukrainian FDs.

Study A highlighted that the interactive training of FDs appeared an effective method that significantly increased knowledge and motivation of FDs to adopt the evidence-based strategy of prevention and cardiovascular risks screening into their clinical practice just after training [20].

Our significant increase of knowledge and motivation confirms the results of a similarly designed Spanish study [24]. The effect of an interactive course including four half-day sessions to develop EBP, was assessed but without long-term follow-up beyond the directly administered questionnaire at the end of the training course. A Chinese study, based on a weekly face-to-face EBP training course, also increased knowledge and future anticipated use [25].

Though we followed the recommendations of a manual for MI training [26], we must be aware that participants can self-report increased competencies 3 months after a course, without an objective change in their capabilities [27].

University teaching and research are key elements of professional empowerment. Since the new research topic *“prevention and screening in FM”* has been suggested to young scientists of our FM department, scientific projects in that field increased. The new topic *“EBS in FM”* was implemented in the teaching program of our University. Knowledge about EBS and motivation to integrate it into the clinical practice, have been implemented.

Study B was original to determine their favorite way to learn about their health or illness. According to Messaadi, using Internet and its digital information are sources of learning about one’s illness, but also about its management and interactions with HCP [28]. Citizens are already actively involved in EBS, using the Internet.

Furthermore, citizens will be empowered by the SA as a shared decision aid tool, based on principles of a motivational interview (MI). According to Miller, MI are underused in daily clinical practice and can increase citizens’ health screening uptake [29]. These findings are confirmed by Broca [30] regarding colon cancer and by Zolfarghi [31] for cervix cancer screening. The adviser triggers the citizens’ active role in their own screening pathway, as they are invited to answer the same question to give their opinion, about their personal intended screening program, before and after the adviser’s suggestions which is a real health democratic and bottom-up strategy to involve citizens in their screening organization. The SA could even be considered as a digital health coach, promoting shared decision making and behavioral changes of the population before the official state based screening program is implemented in Ukraine [32]. Free access to the online *“Screening-adviser”* could increase the awareness of the Ukrainian population about the importance of a future state-based EBS program to implement. Empowered that way, their presently self -selected and composed screening programs would be more evidence-based and efficient during the period without existent EBS in their country.

### Weaknesses (internal, negative factors)

The lack of national guidelines for EBS generates heterogeneous screening attitudes of both, FDs and citizens. Since the Ukrainian Ministry of Health cancelled the prior post-Soviet system of annual check-ups, GPs are allowed to use international EBS guidelines in their own practices which leads to their heterogeneous management. As no gate-keeping system exists yet, citizens are free to choose any investigations they consider as screening without doctor’s advice. Traditional post-Soviet annual check-ups, sometimes covered by patients or recommended by some family doctors, are still performed. The medical educational system requires global changes [21] and young doctors are not trained to EBM and EBS all over the country, hindering citizens’ EBS empowerment.

Without a sustainable national EBS program, motivation and knowledge are likely to decrease, leading to a heterogeneous management of EBS by the FD [20]. Regarding the tendency of loss of motivation in our study A, we suspect the following possible intrinsic factors to explain that phenomenon: personal beliefs, experiences, and professional burnout. Possible extrinsic factors were an inappropriate management and support of FM clinics by the government, and a probable lack of incentives for performance, but further studies are needed to confirm or not that hypothesis.

It is also possible that the motivation decreased due to the difficulty to apply EBP on the long-term [33, 34].

The WHO is an important key partner for the SE as it supports the ongoing health system transformation in Ukraine since 2016 and especially PHC and EBM but EBS is yet not addressed [35]. At present any collaboration between the government and the FDs’ community concerning EBS exists. The reports of the FD national conferences have not been considered by the Ukrainian Ministry of Health which nevertheless made some steps forward EBS since 2017.

### Opportunities (external, positive conditions)

The present lack of national EBS guidelines can also be considered as an opportunity because reliable EBS programs exist all over Europe and can be implemented. WHO is already working with WONCA to promote PHC in Ukraine [35]. The national EBS implementation can be based on European high levels of evidence.

The implementation of a modern, state organized and supported, efficient and effective EBS could foster health democracy and reduce the existent disparities regarding EBS access for common diseases, but implementation of nationwide sustainable EBS requires time, financing and education.

### Threats (external, negative conditions)

The ongoing COVID 19 pandemic accentuates the vulnerable socio-economic situation, hindering a successful implementation of all international EBS strategies in a close future.

No funding can be expected from the government to support state EBS in close future. The ongoing war in the East of Ukraine remains a national priority and requests huge funding, partially alimented by a military support tax corresponding to 1.5% of each citizen’s salary. EBS is not a national priority at present.

### Strengths and limitations

That ongoing implementation study is original because a “bottom-up” or “inside-out”- approach for EBS implementation is used. Using SWOT-analysis to improve quality of healthcare management or implementation studies is original and appropriate [36], especially during an ongoing process like the transition of the PHC-system in Ukraine. The study about the screening adviser is still ongoing and we can’t anticipate yet the next steps to organize first screening of prevalent conditions met in the FD’s office on a local, regional or even national level.

## Conclusions

Ukrainian PHC is in the beginning of transformation. A state based EBS has not been developed yet, but first steps forward were already made. We started EBS implementation through four researches and evaluated their effectiveness, based on a multidimensional empowerment of citizens, HCP and in EBS pathways involved stakeholder teams, to foster a sustainable operational human resource to get involved in that new EBS pathway to implement. The presented SWOT-analysis of this ongoing implementation process allows to plan and optimize future steps towards a state based and supports EBS program in Ukraine.

## Data Availability

Only summarized data sets, generated or analyzed during the current study, are included in this article.

## Abbreviations

BP: Blood Pressure
BMI: Body Mass Index;
EBM: Evidence Based Medicine
EBP: Evidence Based Practice
EBS: Evidence Based Screening
ELISA: Enzyme-Linked Immunosorbent Assay
FD: Family Doctor
FM: Family Medicine
FOBT: Faecal Occult Blood Test
HC: Health Care
HCP: Health Care Professional
HBV/HBC: Hepatitis B/C Virus
HIV: Human Immunodeficiency Virus
HPV: Human Papilloma Virus
MI: Motivational Interview
PCR: Polymerase Chain Reaction
PHC: Primary Health Care
SA: Screening Advisor
SE: State Empowerment
STI: Sexual Transmitted Disease
USPSTF: U.S. Preventive Service Task Force
WHO: World Health Organization
WONCA: World Organization od National Academies and Colleges.

## References

[1] Country and Lending Groups (2019). The World Bank.

[2] Romaniuk P, Semigina T (2018). Ukrainian health care system and its chances for successful transition from soviet legacies. Glob Health.

[3] Health 2020. A European policy framework and strategy for the 21st century / [World Health Organization]. - Copenhagen: WHO regional Office for Europe, 2013. - 181 p.

[4] Lehan VM, Kryachkova LV, Borvinko EV, Volchek VV (2016). Analysis of obstacles to the development of the primary care system in Ukraine and possible approaches to overcome them. Med Perspect.

[5] Lehan VM, Kryachkova LV, Kolesnik VI (2015). Evaluation of the role of primary care physicians in solving common medical problems of citizens on the basis of analysis of medical activity profiles. Modern Med Tech.

[6] Mola E, Eiksson T, Ortiz Bueon MJ, et al. The European definition of general practice/family medicine. 2011Short version . Available at: https://www.woncaeurope.org.

[7] Lehan VM, Kryachkova LV, Gritsenko LO (2017). Evidence-based prevention in the work of a general practitioner - family doctor. Modern Med Tech.

[8] Samokhodsky VM, Smirnova VL, NYa P, Golyachenko BA (2019). Algorithm for realization of organizational and economic prerequisites for evidence-based screening and medical management within healthcare institutions of three levels. Bull Soc Hygiene Health Care Organ Ukraine.

[9] Lehan VM, Kryachkova LV, Maximenko OP (2017). Comparative analysis of approaches to prevention in Europe and Ukraine.

[10] Mikhailovich Y. Problems of adaptation of screening of the most widespread malignancies in the population of Ukraine from the point of view of evidential medicine (lessons of international experience). Clin Oncol. 2012;6(2).

[11] Screening in primary care (2018). Evidence-based clinical guidance. Kyiv.

[12] Lehan VM, Kryachkova LV, Kolesnik VI, Gritsenko CO (2018). The role of the primary link in the organization of evidence-based preventive measures. Fam Med.

[13] Ustinov OV (2013). Primary health care reform - 2013: current state, problems, ways to solve. Ukrainian Med J.

[14] Laws of Ukraine of the Verkhovna Rada of Ukraine. Available at: https://zakon.rada.gov.ua/laws/show/z0846-07.

[15] Canadian Public Health Association (1986). Health and Welfare Canada, World Health Organization. Ottawa Charter for Health Promotion.

[16] U.S. Preventive Service Task Force. https://www.uspreventiveservicestaskforce.org.

[17] Miller WL, Rubinstein EB, Howard J, Crabtree BF (2019). Shifting implementation science theory to empower primary care practices. Ann Fam Med.

[18] Wallerstein N (1992). Powerlessness, empowerment, and health: implications for health promotion programs. Am J Health Promot.

[19] Public Health Quality Improvement Encyclopedia (2012). Public Health Foundation.

[20] Shushman I, Kolesnyk P, Schonmann Y, Harris M, Frese T (2020). Training family doctors and primary care nurses in evidence-based prevention, screening and Management of Cardiovascular Risks in Western Ukraine: a longitudinal study. Zdr Varst.

[21] Kolesnyk P, Svab I (2013). Development of family medicine in Ukraine. Eur J Gen Pract.

[22] Donahue KE, Halladay JR, Wise A (2013). Facilitators of transforming primary care: a look under the Hood at practice leadership. Ann Fam Med.

[23] Israeli Task force for preventive medicine of the Israeli Association of Family Physicians. Available at: website: http://www.shavebdika.org.il/MainPage2.aspx.

[24] Argimon-Pallàs JM, Flores-Mateo G, Jiménez-Villa J, Pujol-Ribera E (2011). Effectiveness of a short-course in improving knowledge and skills on evidence-based practice. BMC Fam Pract.

[25] Fei J, Li Y, Gao W, Li J (2018). Efficacy of evidence-based medicine training for primary healthcare professionals: a non-randomized controlled trial. BMC Med Educ.

[26] Yahne K, Rogers CR, Russel DE (2014). Readiness Ruler Lineup. Motivational interviewing training new trainers manual.

[27] Mash R, Blitz J, Edwards J, Mowle S (2018). Training of workplace-based clinical trainers in family medicine, South Africa: before-and-after evaluation. Afr J Prim Health Care Fam Med.

[28] Messaadi N (2016). Information numérique et gestion de la maladie [Digital data and disease management]. Sante Publique.

[29] Miller SJ, Foran-Tuller K, Ledergerber J (2017). Motivational interviewing to improve health screening uptake: a systematic review. Citizen Educ Counseling.

[30] Broca G, Denis B, Ganaa K (2015). Impact of the telephone motivational interviewing on the colorectal cancer screening participation. A randomized controlled study. Rev Eur Psychol Appl.

[31] Zolfaghari Z, Rezaee N, Shakiba M (2018). Motivational interviewing -based training vs traditional training on the uptake of cervical screening: a quasi-experimental study. Public Health.

[32] Thom DH, Wolf J, Gardner H, Denise DeVore D (2016). A qualitative study of how health coaches support citizens in making health-related decisions and behavioral changes. Ann Fam Med.

[33] Solomons NM, Manag SJAJN (2011). Evidence-based practice barriers and facilitators from a continuous quality improvement perspective: an integrative review. J Nurs Manag.

[34] van Dijk N, Hooft L, Wieringa-de Waard M (2010). What are the barriers to residents’ practicing evidence-based medicine?: a systematic review. Acad Med.

[35] WHO support for health system development in Ukraine, 2016–2019. Copenhagen: WHO Regional Office for Europe; 2019.

[36] Durme V (2014). Stakeholders’ perception on the organization of chronic care: a SWOT analysis to draft avenues for health care reforms. BMC Health Serv Res.

